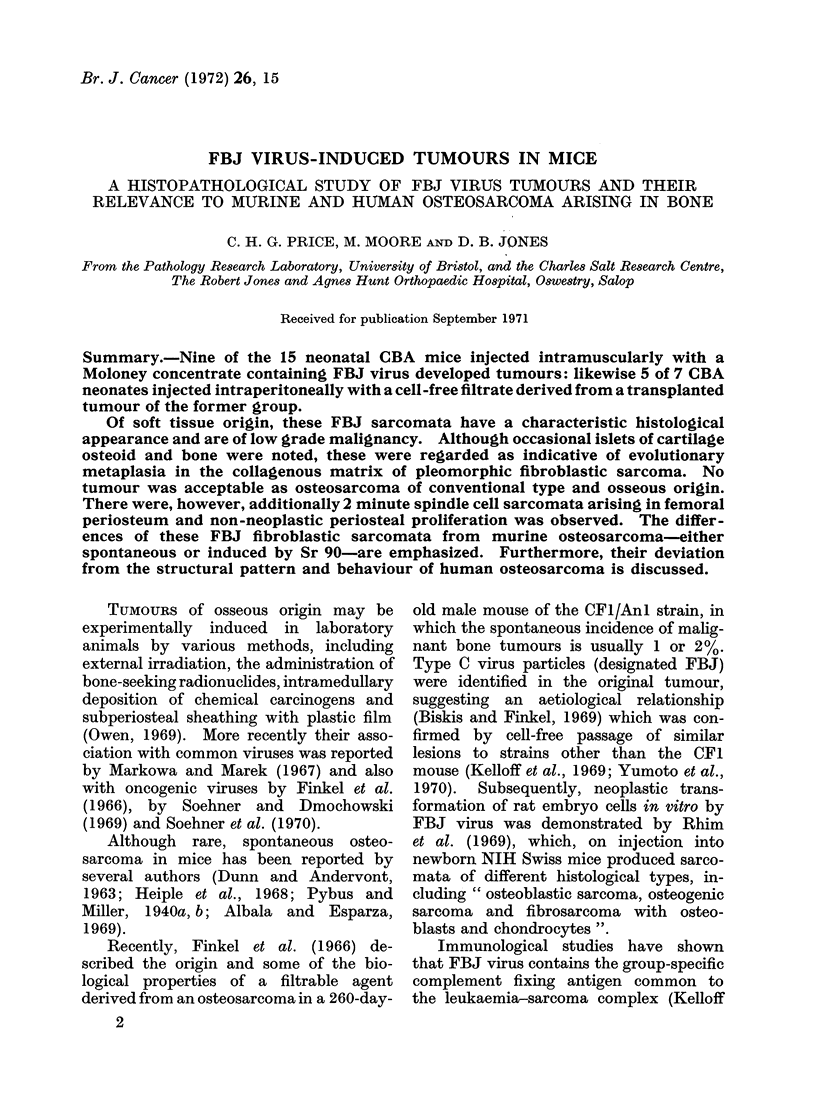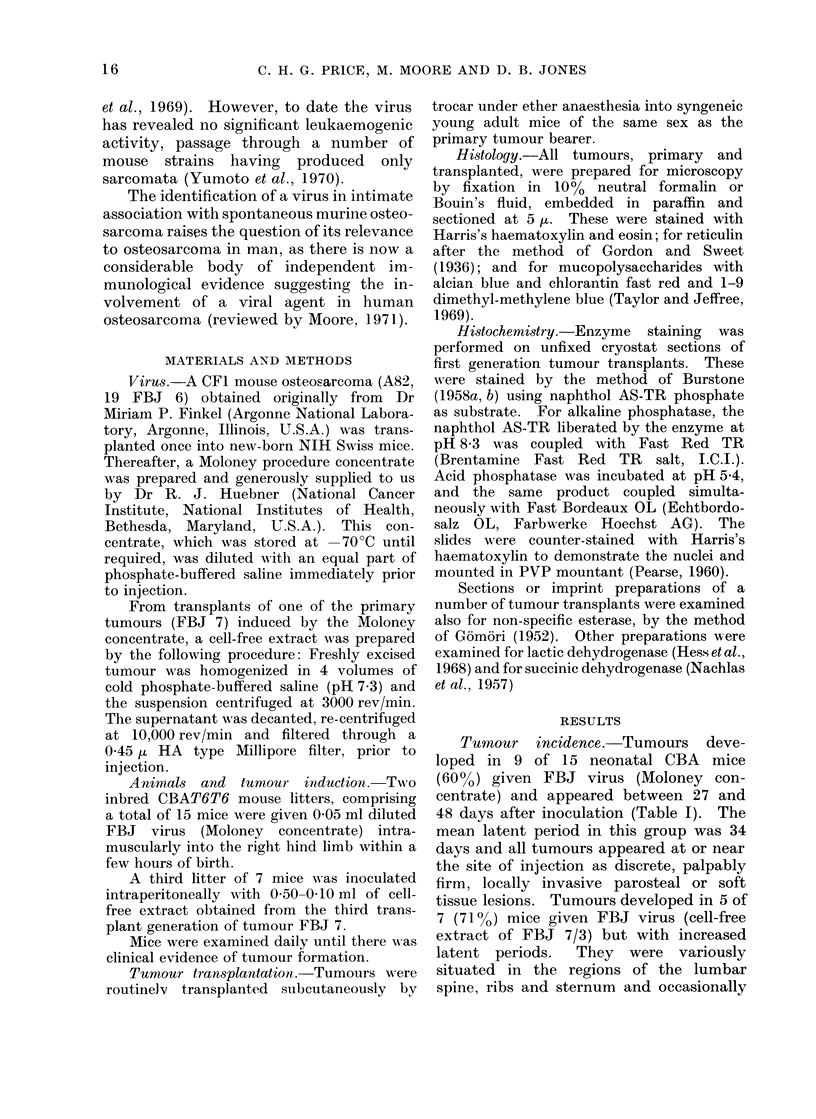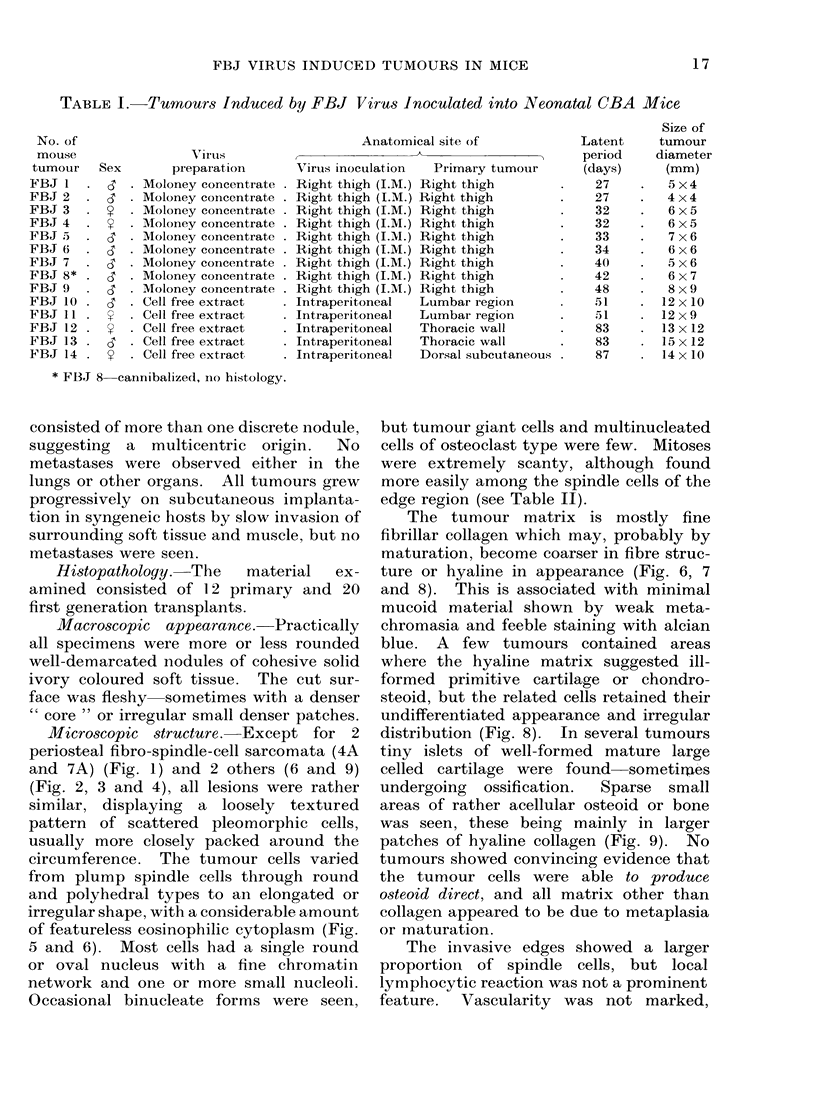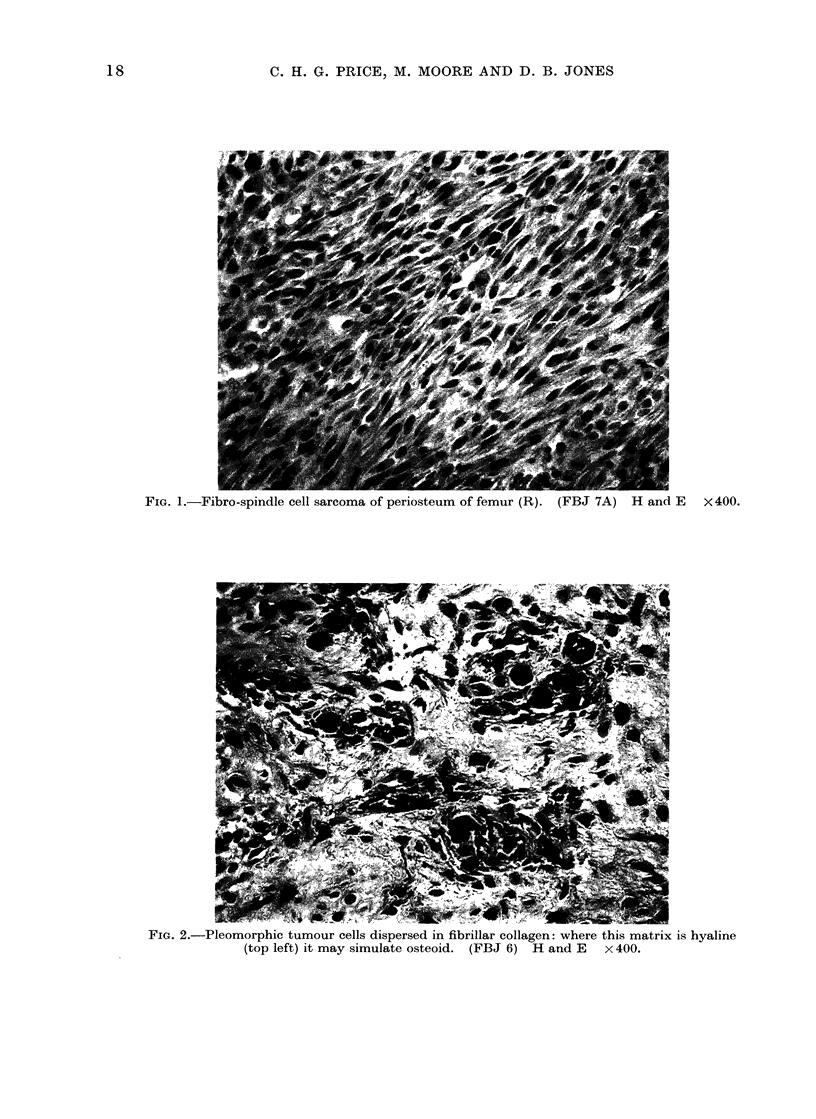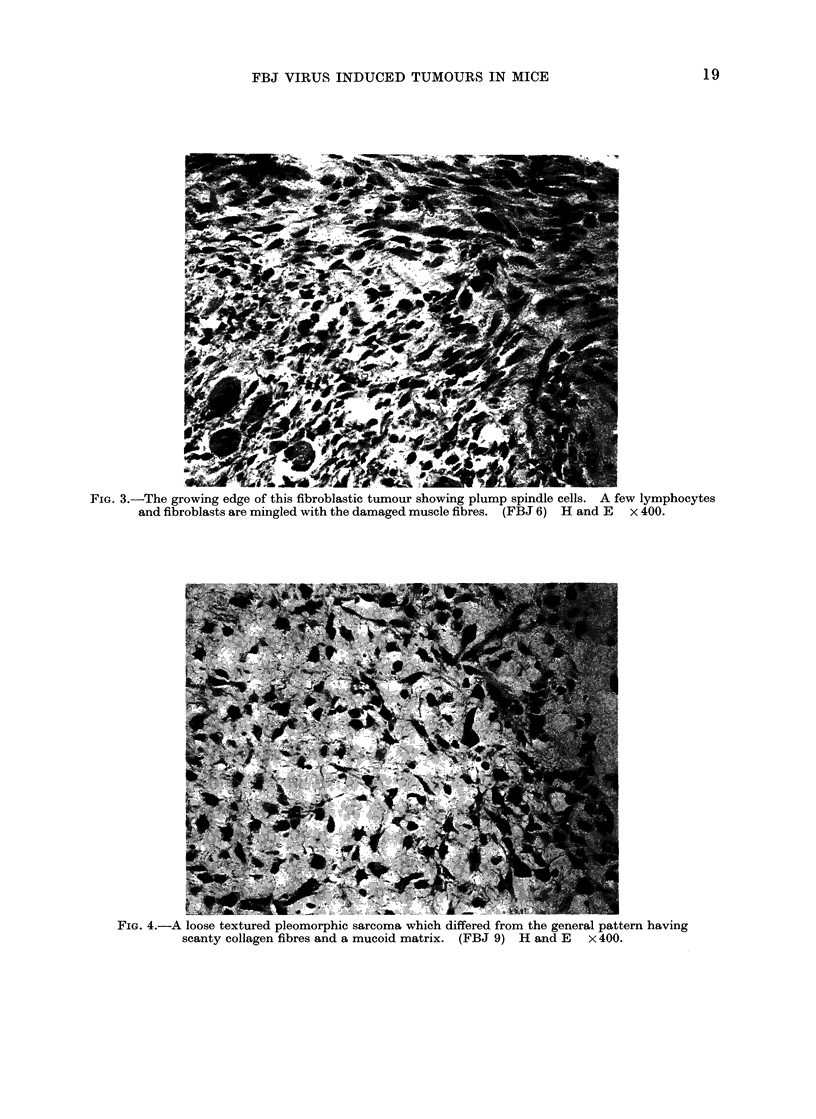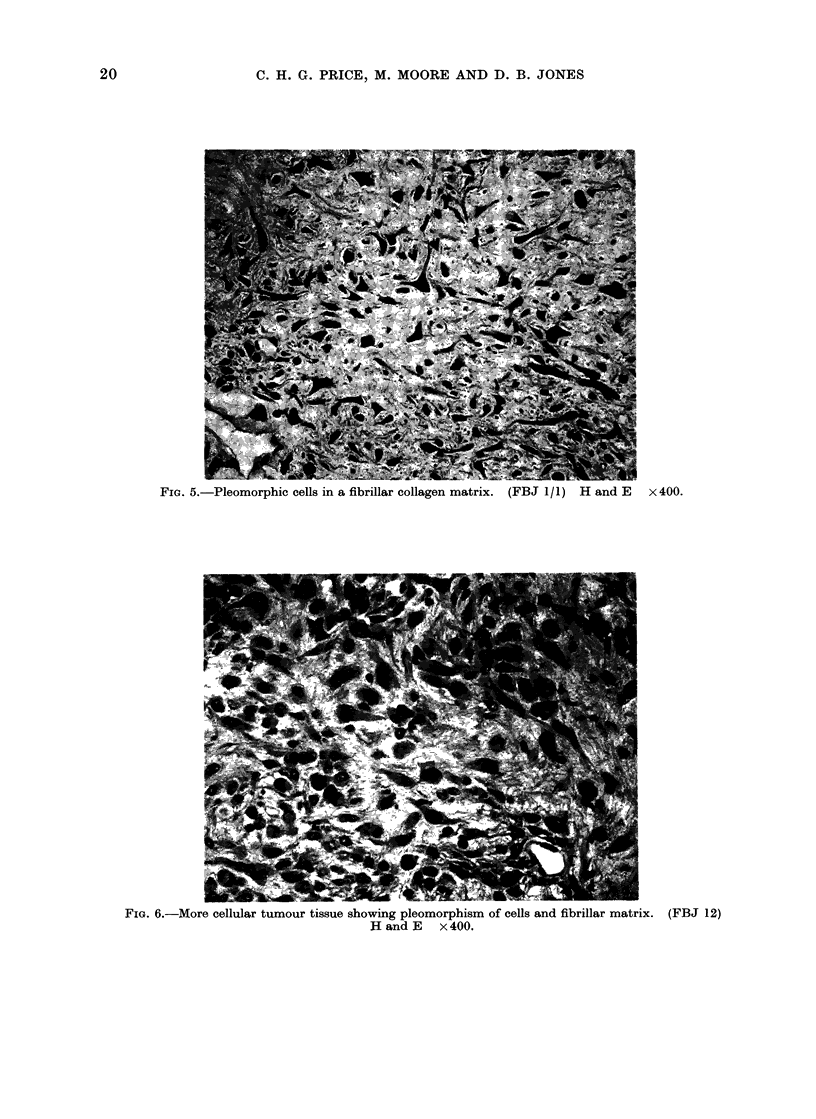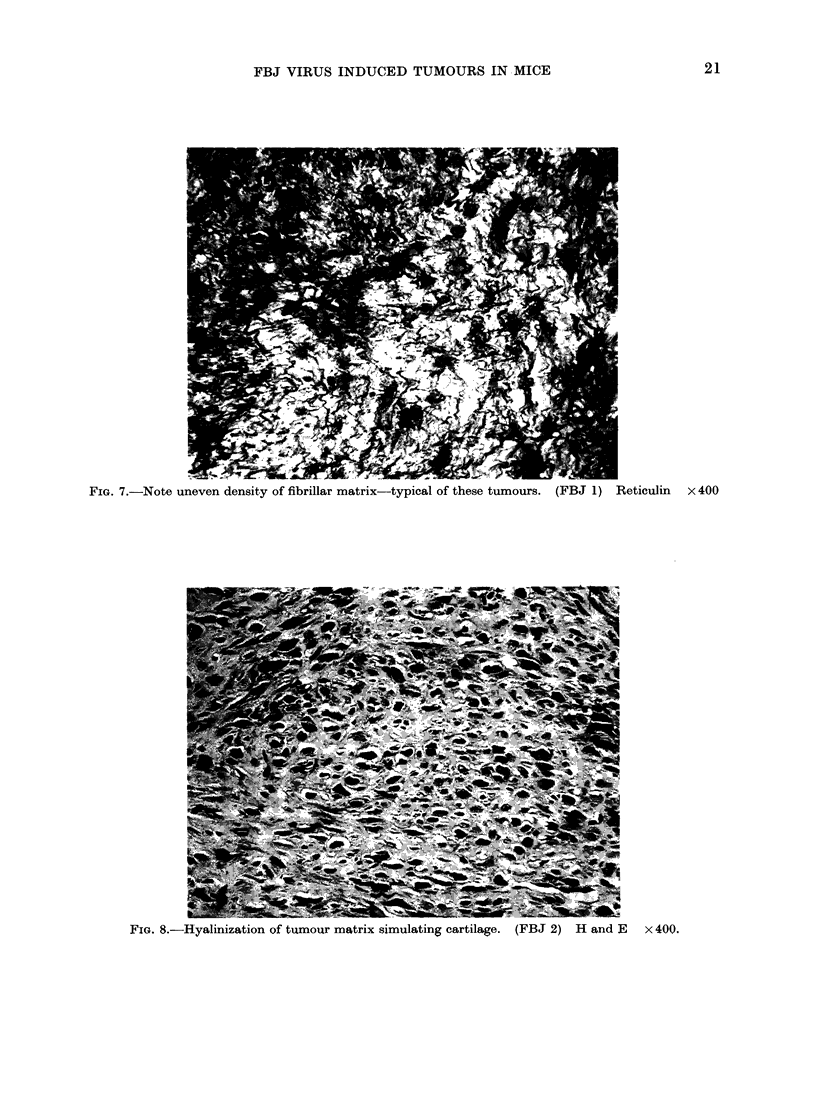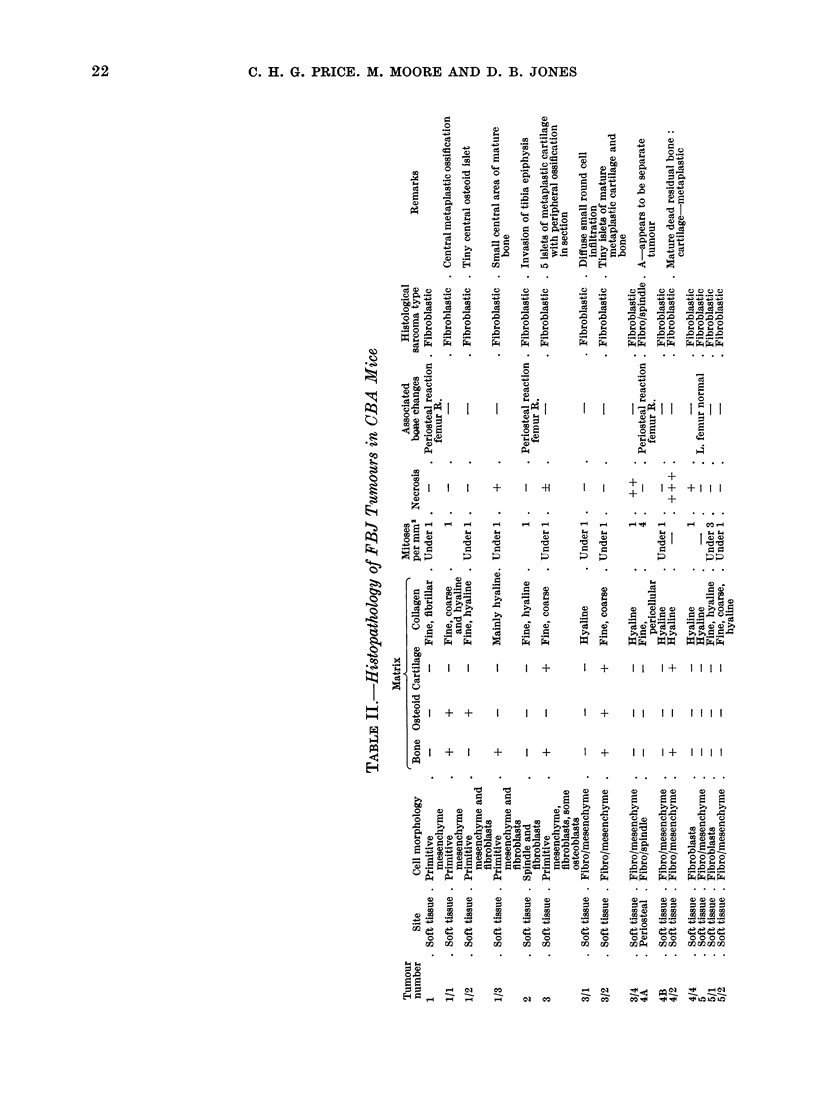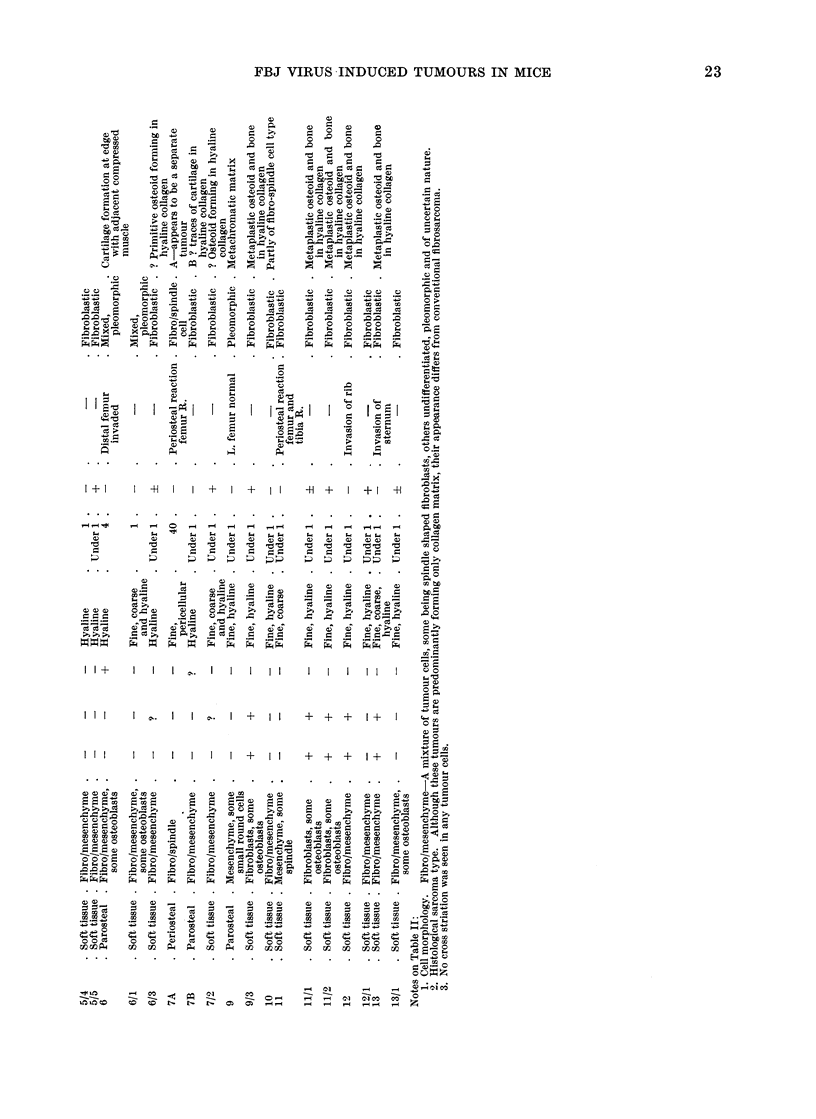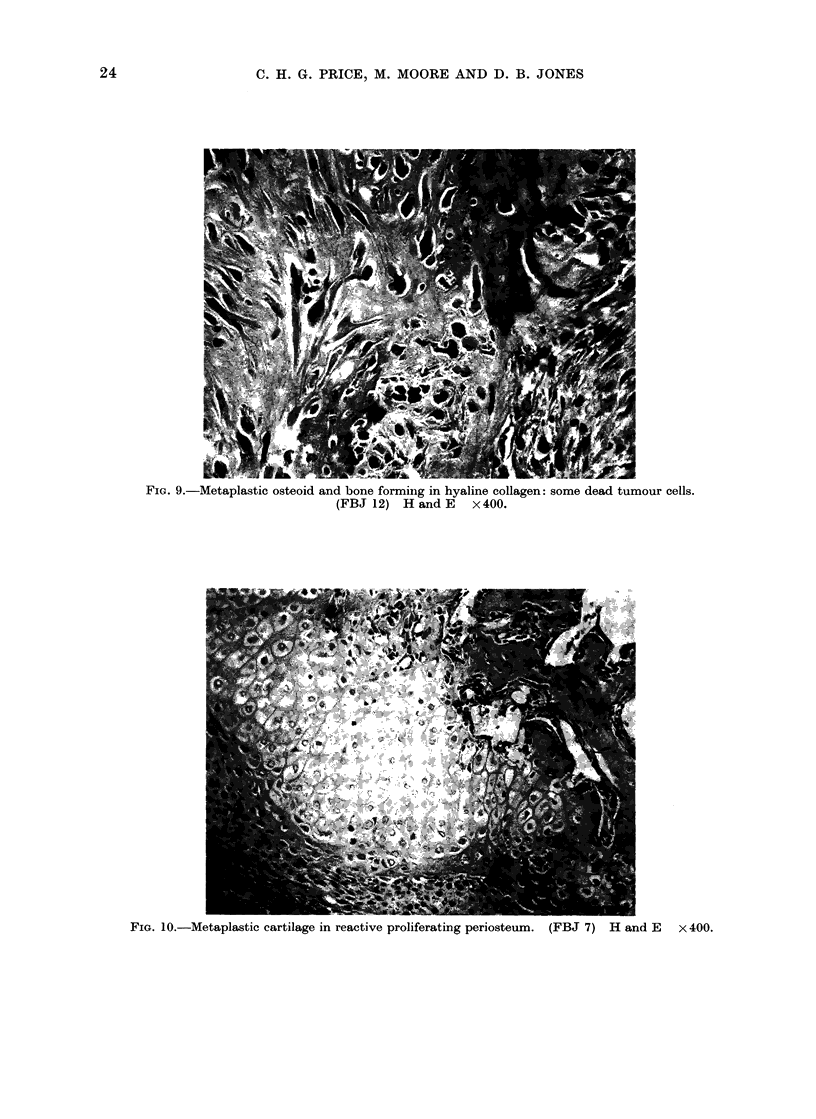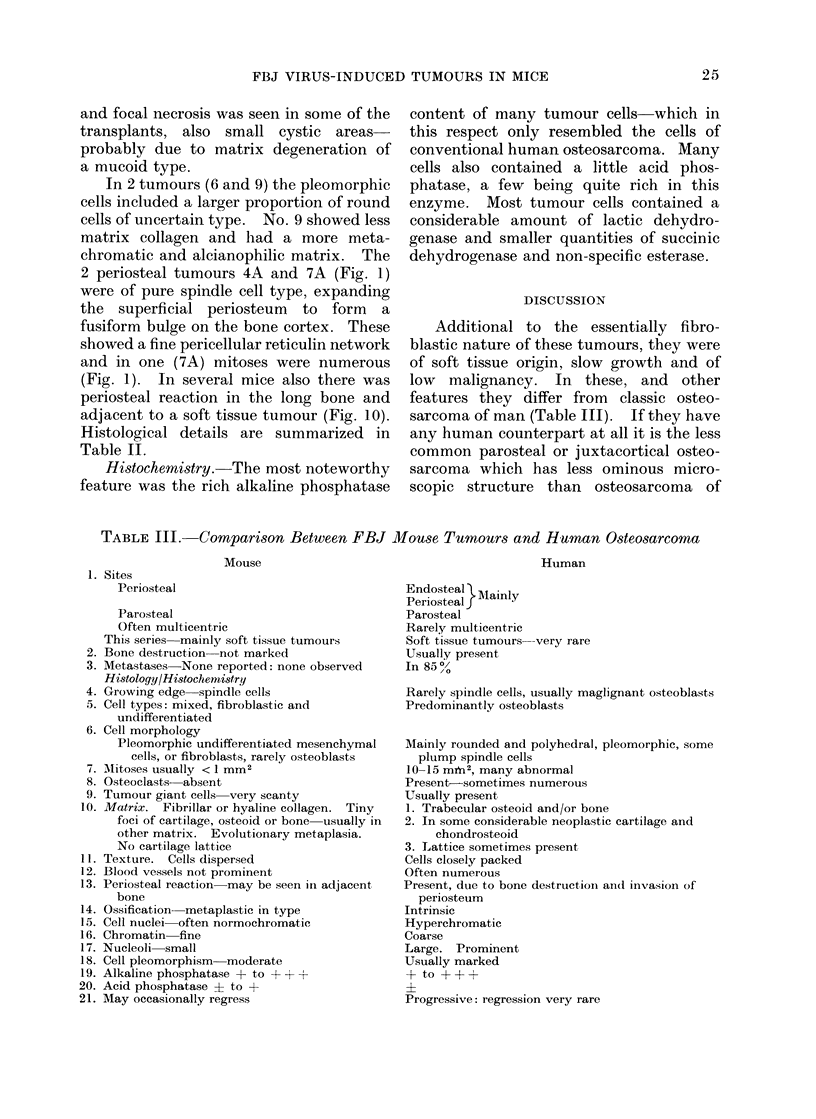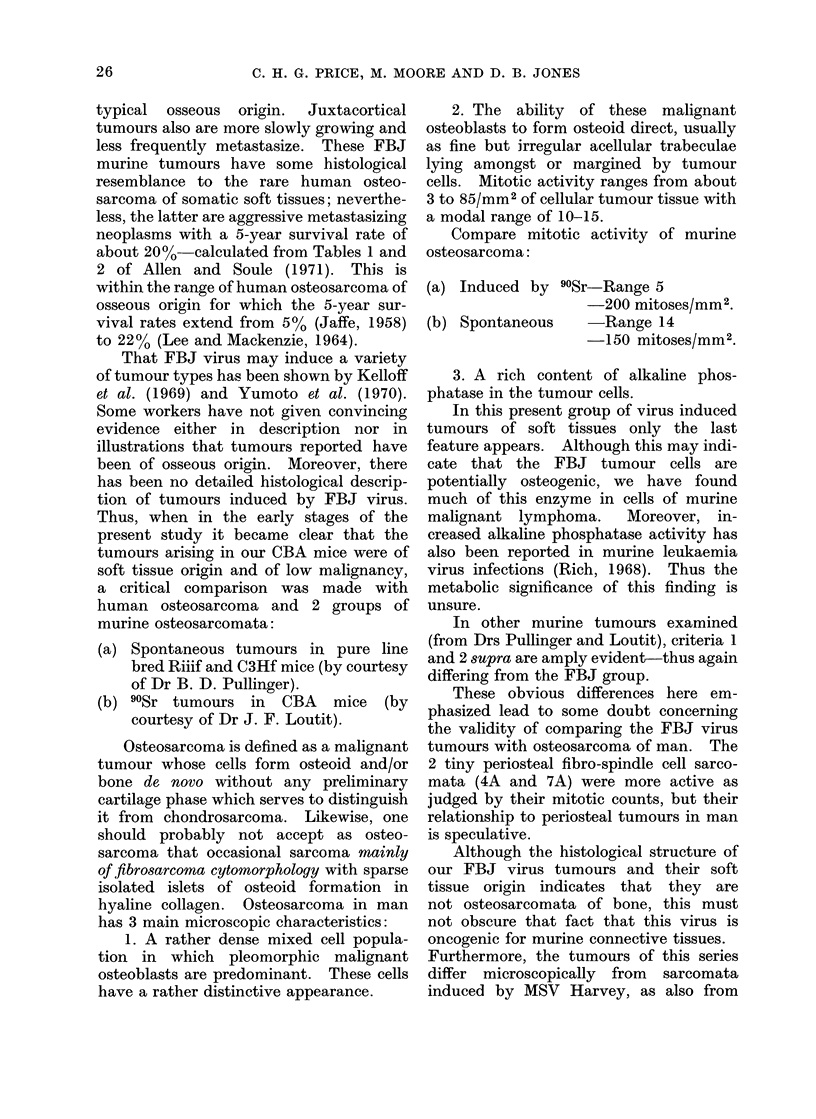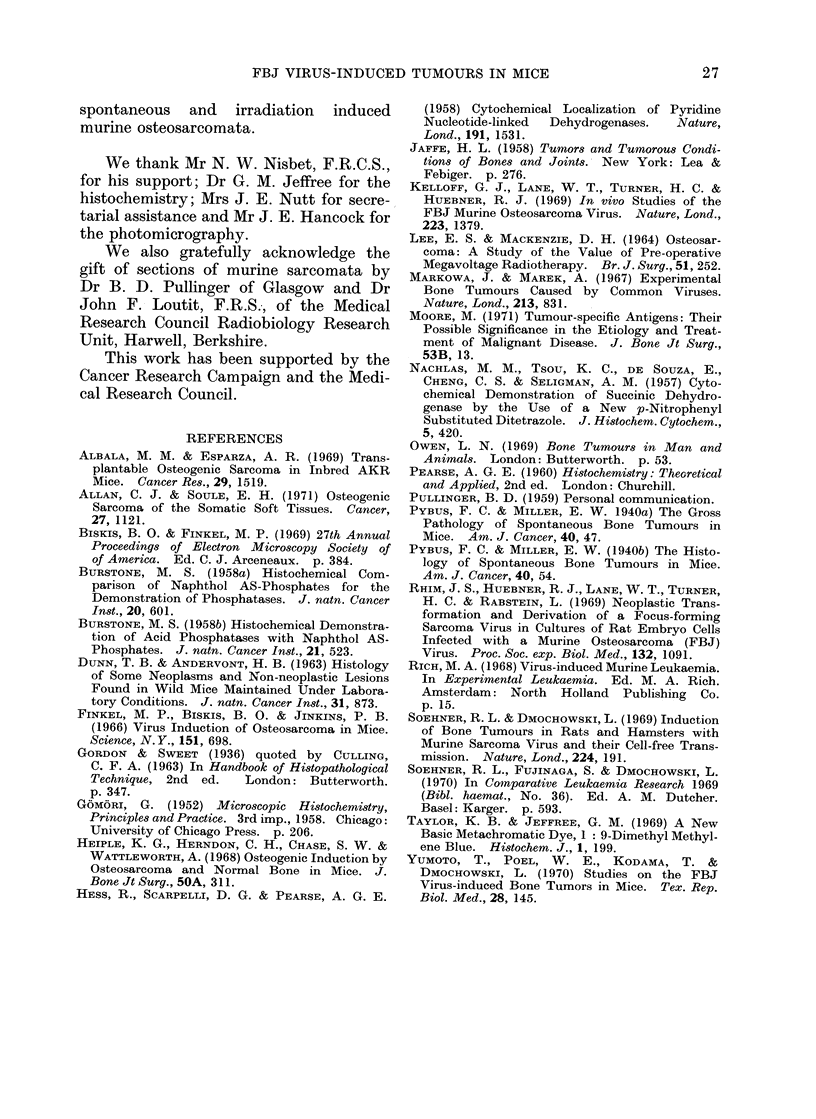# FBJ Virus-induced Tumours in Mice

**DOI:** 10.1038/bjc.1972.5

**Published:** 1972-02

**Authors:** C. H. G. Price, M. Moore, D. B. Jones

## Abstract

**Images:**


*Br. J. Cancer* (1972) **26**, 15

### FBJ VIRUS-INDUCED TUMOURS IN MICE

### A HISTOPATHOLOGICAL STUDY OF FBJ VIRUS TUMOURS AND THEIR RELEVANCE TO MURINE AND HUMAN OSTEOSARCOMA ARISING IN BONE

C. H. G. PRICE, M. MOORE AND D. B. JONES

*From the Pathology Research Laboratory, University of Bristol, and the Charles Salt Research Centre, The Robert Jones and Agnes Hunt Orthopaedic Hospital, Oswestry, Salop*

Received for publication September 1971

**Summary.—Nine of the 15 neonatal CBA mice injected intramuscularly with a Moloney concentrate containing FBJ virus developed tumours: likewise 5 of 7 CBA neonates injected intraperitoneally with a cell-free filtrate derived from a transplanted tumour of the former group.**

**Of soft tissue origin, these FBJ sarcomata have a characteristic histological appearance and are of low grade malignancy. Although occasional islets of cartilage osteoid and bone were noted, these were regarded as indicative of evolutionary metaplasia in the collagenous matrix of pleomorphic fibroblastic sarcoma. No tumour was acceptable as osteosarcoma of conventional type and osseous origin. There were, however, additionally 2 minute spindle cell sarcomata arising in femoral periosteum and non-neoplastic periosteal proliferation was observed. The differences of these FBJ fibroblastic sarcomata from murine osteosarcoma—either spontaneous or induced by Sr 90—are emphasized. Furthermore, their deviation from the structural pattern and behaviour of human osteosarcoma is discussed.**

TUMOURS of osseous origin may be experimentally induced in laboratory animals by various methods, including external irradiation, the administration of bone-seeking radionuclides, intramedullary deposition of chemical carcinogens and subperiosteal sheathing with plastic film (Owen, 1969). More recently their association with common viruses was reported by Markowa and Marek (1967) and also with oncogenic viruses by Finkel *et al.* (1966), by Soehner and Dmochowski (1969) and Soehner *et al.* (1970).

Although rare, spontaneous osteosarcoma in mice has been reported by several authors (Dunn and Andervont, 1963; Heiple *et al.*, 1968; Pybus and Miller, 1940*a*, *b*; Albala and Esparza, 1969).

Recently, Finkel *et al.* (1966) described the origin and some of the biological properties of a filtrable agent derived from an osteosarcoma in a 260-day-old male mouse of the CF1/An1 strain, in which the spontaneous incidence of malignant bone tumours is usually 1 or 2%. Type C virus particles (designated FBJ) were identified in the original tumour, suggesting an aetiological relationship (Biskis and Finkel, 1969) which was confirmed by cell-free passage of similar lesions to strains other than the CF1 mouse (Kelloff *et al.*, 1969; Yumoto *et al.*, 1970). Subsequently, neoplastic transformation of rat embryo cells *in vitro* by FBJ virus was demonstrated by Rhim *et al.* (1969), which, on injection into newborn NIH Swiss mice produced sarcomata of different histological types, including " osteoblastic sarcoma, osteogenic sarcoma and fibrosarcoma with osteoblasts and chondrocytes ".

Immunological studies have shown that FBJ virus contains the group-specific complement fixing antigen common to the leukaemia–sarcoma complex (Kelloff

*et al.*, 1969). However, to date the virus has revealed no significant leukaemogenic activity, passage through a number of mouse strains having produced only sarcomata (Yumoto *et al.*, 1970).

The identification of a virus in intimate association with spontaneous murine osteosarcoma raises the question of its relevance to osteosarcoma in man, as there is now a considerable body of independent immunological evidence suggesting the involvement of a viral agent in human osteosarcoma (reviewed by Moore, 1971).

### MATERIALS AND METHODS

*Virus.*—A CF1 mouse osteosarcoma (A82, 19 FBJ 6) obtained originally from Dr Miriam P. Finkel (Argonne National Laboratory, Argonne, Illinois, U.S.A.) was transplanted once into new-born NIH Swiss mice. Thereafter, a Moloney procedure concentrate was prepared and generously supplied to us by Dr R. J. Huebner (National Cancer Institute, National Institutes of Health, Bethesda, Maryland, U.S.A.). This concentrate, which was stored at —70°C until required, was diluted with an equal part of phosphate-buffered saline immediately prior to injection.

From transplants of one of the primary tumours (FBJ 7) induced by the Moloney concentrate, a cell-free extract was prepared by the following procedure: Freshly excised tumour was homogenized in 4 volumes of cold phosphate-buffered saline (pH 7·3) and the suspension centrifuged at 3000 rev/min. The supernatant was decanted, re-centrifuged at 10,000 rev/min and filtered through a 0·45 $\mu$ HA type Millipore filter, prior to injection.

*Animals and tumour induction.*—Two inbred CBA*T6T6* mouse litters, comprising a total of 15 mice were given 0·05 ml diluted FBJ virus (Moloney concentrate) intramuscularly into the right hind limb within a few hours of birth.

A third litter of 7 mice was inoculated intraperitoneally with 0·50–0·10 ml of cell-free extract obtained from the third transplant generation of tumour FBJ 7.

Mice were examined daily until there was clinical evidence of tumour formation.

*Tumour transplantation.*—Tumours were routinely transplanted subcutaneously by trocar under ether anaesthesia into syngeneic young adult mice of the same sex as the primary tumour bearer.

*Histology.*—All tumours, primary and transplanted, were prepared for microscopy by fixation in 10% neutral formalin or Bouin's fluid, embedded in paraffin and sectioned at 5 $\mu$. These were stained with Harris's haematoxylin and eosin; for reticulin after the method of Gordon and Sweet (1936); and for mucopolysaccharides with alcian blue and chlorantin fast red and 1–9 dimethyl-methylene blue (Taylor and Jeffree, 1969).

*Histochemistry.*—Enzyme staining was performed on unfixed cryostat sections of first generation tumour transplants. These were stained by the method of Burstone (1958*a*, *b*) using naphthol AS-TR phosphate as substrate. For alkaline phosphatase, the naphthol AS-TR liberated by the enzyme at pH 8·3 was coupled with Fast Red TR (Brentamine Fast Red TR salt, I.C.I.). Acid phosphatase was incubated at pH 5·4, and the same product coupled simultaneously with Fast Bordeaux OL (Echtbordosalz OL, Farbwerke Hoechst AG). The slides were counter-stained with Harris's haematoxylin to demonstrate the nuclei and mounted in PVP mountant (Pearse, 1960).

Sections or imprint preparations of a number of tumour transplants were examined also for non-specific esterase, by the method of Gömöri (1952). Other preparations were examined for lactic dehydrogenase (Hess *et al.*, 1968) and for succinic dehydrogenase (Nachlas *et al.*, 1957)

### RESULTS

*Tumour incidence.*—Tumours developed in 9 of 15 neonatal CBA mice (60%) given FBJ virus (Moloney concentrate) and appeared between 27 and 48 days after inoculation (Table I). The mean latent period in this group was 34 days and all tumours appeared at or near the site of injection as discrete, palpably firm, locally invasive parosteal or soft tissue lesions. Tumours developed in 5 of 7 (71%) mice given FBJ virus (cell-free extract of FBJ 7/3) but with increased latent periods. They were variously situated in the regions of the lumbar spine, ribs and sternum and occasionally

TABLE I.—*Tumours Induced by FBJ Virus Inoculated into Neonatal CBA Mice*

| No. of mouse tumour | Sex | Virus preparation | Anatomical site of: Virus inoculation | Anatomical site of: Primary tumour | Latent period (days) | Size of tumour diameter (mm) |
|---|---|---|---|---|---|---|
| FBJ 1 | ♂ | Moloney concentrate | Right thigh (I.M.) | Right thigh | 27 | 5×4 |
| FBJ 2 | ♂ | Moloney concentrate | Right thigh (I.M.) | Right thigh | 27 | 4×4 |
| FBJ 3 | ♀ | Moloney concentrate | Right thigh (I.M.) | Right thigh | 32 | 6×5 |
| FBJ 4 | ♀ | Moloney concentrate | Right thigh (I.M.) | Right thigh | 32 | 6×5 |
| FBJ 5 | ♂ | Moloney concentrate | Right thigh (I.M.) | Right thigh | 33 | 7×6 |
| FBJ 6 | ♂ | Moloney concentrate | Right thigh (I.M.) | Right thigh | 34 | 6×6 |
| FBJ 7 | ♂ | Moloney concentrate | Right thigh (I.M.) | Right thigh | 40 | 5×6 |
| FBJ 8* | ♂ | Moloney concentrate | Right thigh (I.M.) | Right thigh | 42 | 6×7 |
| FBJ 9 | ♂ | Moloney concentrate | Right thigh (I.M.) | Right thigh | 48 | 8×9 |
| FBJ 10 | ♂ | Cell free extract | Intraperitoneal | Lumbar region | 51 | 12×10 |
| FBJ 11 | ♀ | Cell free extract | Intraperitoneal | Lumbar region | 51 | 12×9 |
| FBJ 12 | ♀ | Cell free extract | Intraperitoneal | Thoracic wall | 83 | 13×12 |
| FBJ 13 | ♂ | Cell free extract | Intraperitoneal | Thoracic wall | 83 | 15×12 |
| FBJ 14 | ♀ | Cell free extract | Intraperitoneal | Dorsal subcutaneous | 87 | 14×10 |

* FBJ 8—cannibalized, no histology.

consisted of more than one discrete nodule, suggesting a multicentric origin. No metastases were observed either in the lungs or other organs. All tumours grew progressively on subcutaneous implantation in syngeneic hosts by slow invasion of surrounding soft tissue and muscle, but no metastases were seen.

*Histopathology.*—The material examined consisted of 12 primary and 20 first generation transplants.

*Macroscopic appearance.*—Practically all specimens were more or less rounded well-demarcated nodules of cohesive solid ivory coloured soft tissue. The cut surface was fleshy—sometimes with a denser " core " or irregular small denser patches.

*Microscopic structure.*—Except for 2 periosteal fibro-spindle-cell sarcomata (4A and 7A) (Fig. 1) and 2 others (6 and 9) (Fig. 2, 3 and 4), all lesions were rather similar, displaying a loosely textured pattern of scattered pleomorphic cells, usually more closely packed around the circumference. The tumour cells varied from plump spindle cells through round and polyhedral types to an elongated or irregular shape, with a considerable amount of featureless eosinophilic cytoplasm (Fig. 5 and 6). Most cells had a single round or oval nucleus with a fine chromatin network and one or more small nucleoli. Occasional binucleate forms were seen, but tumour giant cells and multinucleated cells of osteoclast type were few. Mitoses were extremely scanty, although found more easily among the spindle cells of the edge region (see Table II).

The tumour matrix is mostly fine fibrillar collagen which may, probably by maturation, become coarser in fibre structure or hyaline in appearance (Fig. 6, 7 and 8). This is associated with minimal mucoid material shown by weak metachromasia and feeble staining with alcian blue. A few tumours contained areas where the hyaline matrix suggested ill-formed primitive cartilage or chondrosteoid, but the related cells retained their undifferentiated appearance and irregular distribution (Fig. 8). In several tumours tiny islets of well-formed mature large celled cartilage were found—sometimes undergoing ossification. Sparse small areas of rather acellular osteoid or bone was seen, these being mainly in larger patches of hyaline collagen (Fig. 9). No tumours showed convincing evidence that the tumour cells were able *to produce osteoid direct*, and all matrix other than collagen appeared to be due to metaplasia or maturation.

The invasive edges showed a larger proportion of spindle cells, but local lymphocytic reaction was not a prominent feature. Vascularity was not marked,

FIG. 1.—Fibro-spindle cell sarcoma of periosteum of femur (R). (FBJ 7A) H and E ×400.

FIG. 2.—Pleomorphic tumour cells dispersed in fibrillar collagen: where this matrix is hyaline (top left) it may simulate osteoid. (FBJ 6) H and E ×400.

FIG. 3.—The growing edge of this fibroblastic tumour showing plump spindle cells. A few lymphocytes and fibroblasts are mingled with the damaged muscle fibres. (FBJ 6) H and E ×400.

FIG. 4.—A loose textured pleomorphic sarcoma which differed from the general pattern having scanty collagen fibres and a mucoid matrix. (FBJ 9) H and E ×400.

Fig. 5.—Pleomorphic cells in a fibrillar collagen matrix. (FBJ 1/1) H and E ×400.

Fig. 6.—More cellular tumour tissue showing pleomorphism of cells and fibrillar matrix. (FBJ 12) H and E ×400.

FIG. 7.—Note uneven density of fibrillar matrix—typical of these tumours. (FBJ 1) Reticulin ×400

FIG. 8.—Hyalinization of tumour matrix simulating cartilage. (FBJ 2) H and E ×400.

TABLE II.—*Histopathology of FBJ Tumours in CBA Mice*

| Tumour number | Site | Cell morphology | Matrix: Bone | Matrix: Osteoid | Matrix: Cartilage | Matrix: Collagen | Mitoses per mm² | Necrosis | Associated bone changes | Histological sarcoma type | Remarks |
|---|---|---|---|---|---|---|---|---|---|---|---|
| 1 | Soft tissue | Primitive mesenchyme | — | — | — | Fine, fibrillar | Under 1 | — | Periosteal reaction femur R. | Fibroblastic | |
| 1/1 | Soft tissue | Primitive mesenchyme | + | + | — | Fine, coarse and hyaline | 1 | — | — | Fibroblastic | Central metaplastic ossification |
| 1/2 | Soft tissue | Primitive mesenchyme and fibroblasts | — | + | — | Fine, hyaline | Under 1 | — | — | Fibroblastic | Tiny central osteoid islet |
| 1/3 | Soft tissue | Primitive mesenchyme and fibroblasts | + | — | — | Mainly hyaline | Under 1 | + | — | Fibroblastic | Small central area of mature bone |
| 2 | Soft tissue | Spindle and fibroblasts | — | — | — | Fine, hyaline | 1 | — | Periosteal reaction femur R. | Fibroblastic | Invasion of tibia epiphysis |
| 3 | Soft tissue | Primitive mesenchyme, fibroblasts, some osteoblasts | + | — | + | Fine, coarse | Under 1 | ± | — | Fibroblastic | 5 islets of metaplastic cartilage with peripheral ossification in section |
| 3/1 | Soft tissue | Fibro/mesenchyme | — | — | — | Hyaline | Under 1 | — | — | Fibroblastic | Diffuse small round cell infiltration |
| 3/2 | Soft tissue | Fibro/mesenchyme | + | + | + | Fine, coarse | Under 1 | — | — | Fibroblastic | Tiny islets of mature metaplastic cartilage and bone |
| 3/4 | Soft tissue | Fibro/mesenchyme | — | — | — | Hyaline | 1 | ++ | — | Fibroblastic | |
| 4A | Periosteal | Fibro/spindle | — | — | — | Fine, pericellular | 4 | — | Periosteal reaction femur R. | Fibro/spindle | A—appears to be separate tumour |
| 4B | Soft tissue | Fibro/mesenchyme | — | — | — | Hyaline | Under 1 | — | — | Fibroblastic | |
| 4/2 | Soft tissue | Fibro/mesenchyme | + | — | + | Hyaline | — | +++ | — | Fibroblastic | Mature dead residual bone : cartilage—metaplastic |
| 4/4 | Soft tissue | Fibroblasts | — | — | — | Hyaline | 1 | + | — | Fibroblastic | |
| 5 | Soft tissue | Fibro/mesenchyme | — | — | — | Hyaline | — | — | L. femur normal | Fibroblastic | |
| 5/1 | Soft tissue | Fibroblasts | — | — | — | Fine, hyaline | Under 3 | — | — | Fibroblastic | |
| 5/2 | Soft tissue | Fibro/mesenchyme | — | — | — | Fine, coarse, hyaline | Under 1 | — | — | Fibroblastic | |

| | | | | | | | | | | | |
|---|---|---|---|---|---|---|---|---|---|---|---|
| 5/4 | Soft tissue | Fibro/mesenchyme | — | — | — | Hyaline | 1 | — | — | Fibroblastic | |
| 5/5 | Soft tissue | Fibro/mesenchyme | — | — | — | Hyaline | Under 1 | + | — | Fibroblastic | |
| 6 | Parosteal | Fibro/mesenchyme, some osteoblasts | — | — | + | Hyaline | 4 | — | Distal femur invaded | Mixed, pleomorphic | Cartilage formation at edge with adjacent compressed muscle |
| 6/1 | Soft tissue | Fibro/mesenchyme, some osteoblasts | — | — | — | Fine, coarse and hyaline | 1 | — | — | Mixed, pleomorphic | |
| 6/3 | Soft tissue | Fibro/mesenchyme | — | ? | — | Hyaline | Under 1 | ± | — | Fibroblastic | ? Primitive osteoid forming in hyaline collagen |
| 7A | Periosteal | Fibro/spindle | — | — | — | Fine, pericellular | 40 | — | Periosteal reaction femur R. | Fibro/spindle cell | A—appears to be a separate tumour |
| 7B | Parosteal | Fibro/mesenchyme | — | — | ? | Hyaline | Under 1 | — | — | Fibroblastic | B ? traces of cartilage in hyaline collagen |
| 7/2 | Soft tissue | Fibro/mesenchyme | — | ? | — | Fine, coarse and hyaline | Under 1 | + | — | Fibroblastic | ? Osteoid forming in hyaline collagen |
| 9 | Parosteal | Mesenchyme, some small round cells | — | — | — | Fine, hyaline | Under 1 | — | L. femur normal | Pleomorphic | Metachromatic matrix |
| 9/3 | Soft tissue | Fibroblasts, some osteoblasts | + | + | — | Fine, hyaline | Under 1 | + | — | Fibroblastic | Metaplastic osteoid and bone in hyaline collagen |
| 10 | Soft tissue | Fibro/mesenchyme | — | — | — | Fine, hyaline | Under 1 | — | — | Fibroblastic | Partly of fibro-spindle cell type |
| 11 | Soft tissue | Mesenchyme, some spindle | — | — | — | Fine, coarse | Under 1 | — | Periosteal reaction femur and tibia R. | Fibroblastic | |
| 11/1 | Soft tissue | Fibroblasts, some osteoblasts | + | + | — | Fine, hyaline | Under 1 | ± | — | Fibroblastic | Metaplastic osteoid and bone in hyaline collagen |
| 11/2 | Soft tissue | Fibroblasts, some osteoblasts | + | + | — | Fine, hyaline | Under 1 | + | — | Fibroblastic | Metaplastic osteoid and bone in hyaline collagen |
| 12 | Soft tissue | Fibro/mesenchyme | + | + | — | Fine, hyaline | Under 1 | — | Invasion of rib | Fibroblastic | Metaplastic osteoid and bone in hyaline collagen |
| 12/1 | Soft tissue | Fibro/mesenchyme | — | — | — | Fine, hyaline | Under 1 | + | — | Fibroblastic | |
| 13 | Soft tissue | Fibro/mesenchyme | + | + | — | Fine, coarse, hyaline | Under 1 | — | Invasion of sternum | Fibroblastic | Metaplastic osteoid and bone in hyaline collagen |
| 13/1 | Soft tissue | Fibro/mesenchyme, some osteoblasts | — | — | — | Fine, hyaline | Under 1 | ± | — | Fibroblastic | |

Notes on Table II:

1. Cell morphology. Fibro/mesenchyme—A mixture of tumour cells, some being spindle shaped fibroblasts, others undifferentiated, pleomorphic and of uncertain nature.

2. Histological sarcoma type. Although these tumours are predominantly forming only collagen matrix, their appearance differs from conventional fibrosarcoma.

3. No cross striation was seen in any tumour cells.

FIG. 9.—Metaplastic osteoid and bone forming in hyaline collagen: some dead tumour cells. (FBJ 12) H and E ×400.

FIG. 10.—Metaplastic cartilage in reactive proliferating periosteum. (FBJ 7) H and E ×400.

and focal necrosis was seen in some of the transplants, also small cystic areas—probably due to matrix degeneration of a mucoid type.

In 2 tumours (6 and 9) the pleomorphic cells included a larger proportion of round cells of uncertain type. No. 9 showed less matrix collagen and had a more metachromatic and alcianophilic matrix. The 2 periosteal tumours 4A and 7A (Fig. 1) were of pure spindle cell type, expanding the superficial periosteum to form a fusiform bulge on the bone cortex. These showed a fine pericellular reticulin network and in one (7A) mitoses were numerous (Fig. 1). In several mice also there was periosteal reaction in the long bone and adjacent to a soft tissue tumour (Fig. 10). Histological details are summarized in Table II.

*Histochemistry.*—The most noteworthy feature was the rich alkaline phosphatase content of many tumour cells—which in this respect only resembled the cells of conventional human osteosarcoma. Many cells also contained a little acid phosphatase, a few being quite rich in this enzyme. Most tumour cells contained a considerable amount of lactic dehydrogenase and smaller quantities of succinic dehydrogenase and non-specific esterase.

### DISCUSSION

Additional to the essentially fibroblastic nature of these tumours, they were of soft tissue origin, slow growth and of low malignancy. In these, and other features they differ from classic osteosarcoma of man (Table III). If they have any human counterpart at all it is the less common parosteal or juxtacortical osteosarcoma which has less ominous microscopic structure than osteosarcoma of

TABLE III.—*Comparison Between FBJ Mouse Tumours and Human Osteosarcoma*

| Mouse | Human |
|---|---|
| 1. Sites | |
| Periosteal | Endosteal<br>Periosteal } Mainly |
| Parosteal | Parosteal |
| Often multicentric | Rarely multicentric |
| This series—mainly soft tissue tumours | Soft tissue tumours—very rare |
| 2. Bone destruction—not marked | Usually present |
| 3. Metastases—None reported: none observed | In 85% |
| *Histology/Histochemistry* | |
| 4. Growing edge—spindle cells | Rarely spindle cells, usually maglignant osteoblasts |
| 5. Cell types: mixed, fibroblastic and undifferentiated | Predominantly osteoblasts |
| 6. Cell morphology | |
| Pleomorphic undifferentiated mesenchymal cells, or fibroblasts, rarely osteoblasts | Mainly rounded and polyhedral, pleomorphic, some plump spindle cells |
| 7. Mitoses usually $< 1$ mm$^2$ | 10–15 mm$^2$, many abnormal |
| 8. Osteoclasts—absent | Present—sometimes numerous |
| 9. Tumour giant cells—very scanty | Usually present |
| 10. *Matrix.* Fibrillar or hyaline collagen. Tiny foci of cartilage, osteoid or bone—usually in other matrix. Evolutionary metaplasia. No cartilage lattice | 1. Trabecular osteoid and/or bone<br>2. In some considerable neoplastic cartilage and chondrosteoid<br>3. Lattice sometimes present |
| 11. Texture. Cells dispersed | Cells closely packed |
| 12. Blood vessels not prominent | Often numerous |
| 13. Periosteal reaction—may be seen in adjacent bone | Present, due to bone destruction and invasion of periosteum |
| 14. Ossification—metaplastic in type | Intrinsic |
| 15. Cell nuclei—often normochromatic | Hyperchromatic |
| 16. Chromatin—fine | Coarse |
| 17. Nucleoli—small | Large. Prominent |
| 18. Cell pleomorphism—moderate | Usually marked |
| 19. Alkaline phosphatase $+$ to $+++$ | $+$ to $+++$ |
| 20. Acid phosphatase $\pm$ to $+$ | $\pm$ |
| 21. May occasionally regress | Progressive: regression very rare |

typical osseous origin. Juxtacortical tumours also are more slowly growing and less frequently metastasize. These FBJ murine tumours have some histological resemblance to the rare human osteosarcoma of somatic soft tissues; nevertheless, the latter are aggressive metastasizing neoplasms with a 5-year survival rate of about 20%—calculated from Tables 1 and 2 of Allen and Soule (1971). This is within the range of human osteosarcoma of osseous origin for which the 5-year survival rates extend from 5% (Jaffe, 1958) to 22% (Lee and Mackenzie, 1964).

That FBJ virus may induce a variety of tumour types has been shown by Kelloff *et al.* (1969) and Yumoto *et al.* (1970). Some workers have not given convincing evidence either in description nor in illustrations that tumours reported have been of osseous origin. Moreover, there has been no detailed histological description of tumours induced by FBJ virus. Thus, when in the early stages of the present study it became clear that the tumours arising in our CBA mice were of soft tissue origin and of low malignancy, a critical comparison was made with human osteosarcoma and 2 groups of murine osteosarcomata:

(a) Spontaneous tumours in pure line bred Riiif and C3Hf mice (by courtesy of Dr B. D. Pullinger).
(b) $^{90}$Sr tumours in CBA mice (by courtesy of Dr J. F. Loutit).

Osteosarcoma is defined as a malignant tumour whose cells form osteoid and/or bone *de novo* without any preliminary cartilage phase which serves to distinguish it from chondrosarcoma. Likewise, one should probably not accept as osteosarcoma that occasional sarcoma *mainly of fibrosarcoma cytomorphology* with sparse isolated islets of osteoid formation in hyaline collagen. Osteosarcoma in man has 3 main microscopic characteristics:

1. A rather dense mixed cell population in which pleomorphic malignant osteoblasts are predominant. These cells have a rather distinctive appearance.

2. The ability of these malignant osteoblasts to form osteoid direct, usually as fine but irregular acellular trabeculae lying amongst or margined by tumour cells. Mitotic activity ranges from about 3 to 85/mm$^2$ of cellular tumour tissue with a modal range of 10–15.

Compare mitotic activity of murine osteosarcoma:

(a) Induced by $^{90}$Sr—Range 5 —200 mitoses/mm$^2$.
(b) Spontaneous —Range 14 —150 mitoses/mm$^2$.

3. A rich content of alkaline phosphatase in the tumour cells.

In this present group of virus induced tumours of soft tissues only the last feature appears. Although this may indicate that the FBJ tumour cells are potentially osteogenic, we have found much of this enzyme in cells of murine malignant lymphoma. Moreover, increased alkaline phosphatase activity has also been reported in murine leukaemia virus infections (Rich, 1968). Thus the metabolic significance of this finding is unsure.

In other murine tumours examined (from Drs Pullinger and Loutit), criteria 1 and 2 *supra* are amply evident—thus again differing from the FBJ group.

These obvious differences here emphasized lead to some doubt concerning the validity of comparing the FBJ virus tumours with osteosarcoma of man. The 2 tiny periosteal fibro-spindle cell sarcomata (4A and 7A) were more active as judged by their mitotic counts, but their relationship to periosteal tumours in man is speculative.

Although the histological structure of our FBJ virus tumours and their soft tissue origin indicates that they are not osteosarcomata of bone, this must not obscure that fact that this virus is oncogenic for murine connective tissues. Furthermore, the tumours of this series differ microscopically from sarcomata induced by MSV Harvey, as also from spontaneous and irradiation induced murine osteosarcomata.

We thank Mr N. W. Nisbet, F.R.C.S., for his support; Dr G. M. Jeffree for the histochemistry; Mrs J. E. Nutt for secretarial assistance and Mr J. E. Hancock for the photomicrography.

We also gratefully acknowledge the gift of sections of murine sarcomata by Dr B. D. Pullinger of Glasgow and Dr John F. Loutit, F.R.S., of the Medical Research Council Radiobiology Research Unit, Harwell, Berkshire.

This work has been supported by the Cancer Research Campaign and the Medical Research Council.

### REFERENCES

ALBALA, M. M. & ESPARZA, A. R. (1969) Transplantable Osteogenic Sarcoma in Inbred AKR Mice. *Cancer Res.*, **29**, 1519.

ALLAN, C. J. & SOULE, E. H. (1971) Osteogenic Sarcoma of the Somatic Soft Tissues. *Cancer*, **27**, 1121.

BISKIS, B. O. & FINKEL, M. P. (1969) *27th Annual Proceedings of Electron Microscopy Society of of America.* Ed. C. J. Arceneaux. p. 384.

BURSTONE, M. S. (1958*a*) Histochemical Comparison of Naphthol AS-Phosphates for the Demonstration of Phosphatases. *J. natn. Cancer Inst.*, **20**, 601.

BURSTONE, M. S. (1958*b*) Histochemical Demonstration of Acid Phosphatases with Naphthol AS-Phosphates. *J. natn. Cancer Inst.*, **21**, 523.

DUNN, T. B. & ANDERVONT, H. B. (1963) Histology of Some Neoplasms and Non-neoplastic Lesions Found in Wild Mice Maintained Under Laboratory Conditions. *J. natn. Cancer Inst.*, **31**, 873.

FINKEL, M. P., BISKIS, B. O. & JINKINS, P. B. (1966) Virus Induction of Osteosarcoma in Mice. *Science, N.Y.*, **151**, 698.

GORDON & SWEET (1936) quoted by CULLING, C. F. A. (1963) In *Handbook of Histopathological Technique*, 2nd ed. London: Butterworth. p. 347.

GÖMÖRI, G. (1952) *Microscopic Histochemistry, Principles and Practice.* 3rd imp., 1958. Chicago: University of Chicago Press. p. 206.

HEIPLE, K. G., HERNDON, C. H., CHASE, S. W. & WATTLEWORTH, A. (1968) Osteogenic Induction by Osteosarcoma and Normal Bone in Mice. *J. Bone Jt Surg.*, **50A**, 311.

HESS, R., SCARPELLI, D. G. & PEARSE, A. G. E. (1958) Cytochemical Localization of Pyridine Nucleotide-linked Dehydrogenases. *Nature, Lond.*, **191**, 1531.

JAFFE, H. L. (1958) *Tumors and Tumorous Conditions of Bones and Joints.* New York: Lea & Febiger. p. 276.

KELLOFF, G. J., LANE, W. T., TURNER, H. C. & HUEBNER, R. J. (1969) *In vivo* Studies of the FBJ Murine Osteosarcoma Virus. *Nature, Lond.*, **223**, 1379.

LEE, E. S. & MACKENZIE, D. H. (1964) Osteosarcoma: A Study of the Value of Pre-operative Megavoltage Radiotherapy. *Br. J. Surg.*, **51**, 252.

MARKOWA, J. & MAREK, A. (1967) Experimental Bone Tumours Caused by Common Viruses. *Nature, Lond.*, **213**, 831.

MOORE, M. (1971) Tumour-specific Antigens: Their Possible Significance in the Etiology and Treatment of Malignant Disease. *J. Bone Jt Surg.*, **53B**, 13.

NACHLAS, M. M., TSOU, K. C., DE SOUZA, E., CHENG, C. S. & SELIGMAN, A. M. (1957) Cytochemical Demonstration of Succinic Dehydrogenase by the Use of a New *p*-Nitrophenyl Substituted Ditetrazole. *J. Histochem. Cytochem.*, **5**, 420.

OWEN, L. N. (1969) *Bone Tumours in Man and Animals.* London: Butterworth. p. 53.

PEARSE, A. G. E. (1960) *Histochemistry: Theoretical and Applied*, 2nd ed. London: Churchill.

PULLINGER, B. D. (1959) Personal communication.

PYBUS, F. C. & MILLER, E. W. 1940*a*) The Gross Pathology of Spontaneous Bone Tumours in Mice. *Am. J. Cancer*, **40**, 47.

PYBUS, F. C. & MILLER, E. W. (1940*b*) The Histology of Spontaneous Bone Tumours in Mice. *Am. J. Cancer*, **40**, 54.

RHIM, J. S., HUEBNER, R. J., LANE, W. T., TURNER, H. C. & RABSTEIN, L. (1969) Neoplastic Transformation and Derivation of a Focus-forming Sarcoma Virus in Cultures of Rat Embryo Cells Infected with a Murine Osteosarcoma (FBJ) Virus. *Proc. Soc. exp. Biol. Med.*, **132**, 1091.

RICH, M. A. (1968) Virus-induced Murine Leukaemia. In *Experimental Leukaemia.* Ed. M. A. Rich. Amsterdam: North Holland Publishing Co. p. 15.

SOEHNER, R. L. & DMOCHOWSKI, L. (1969) Induction of Bone Tumours in Rats and Hamsters with Murine Sarcoma Virus and their Cell-free Transmission. *Nature, Lond.*, **224**, 191.

SOEHNER, R. L., FUJINAGA, S. & DMOCHOWSKI, L. (1970) In *Comparative Leukaemia Research* 1969 (*Bibl. haemat.*, No. 36). Ed. A. M. Dutcher. Basel: Karger. p. 593.

TAYLOR, K. B. & JEFFREE, G. M. (1969) A New Basic Metachromatic Dye, 1 : 9-Dimethyl Methylene Blue. *Histochem. J.*, **1**, 199.

YUMOTO, T., POEL, W. E., KODAMA, T. & DMOCHOWSKI, L. (1970) Studies on the FBJ Virus-induced Bone Tumors in Mice. *Tex. Rep. Biol. Med.*, **28**, 145.